# AI for Ancient Games: Report on the Digital Ludeme Project

**DOI:** 10.1007/s13218-019-00600-6

**Published:** 2019-07-01

**Authors:** Cameron Browne

**Affiliations:** Department of Data Science and Knowledge Engineering (DKE), Maastricht University, Bouillonstraat 8-10, 6211 LH Maastricht, The Netherlands

**Keywords:** Ancient games, General game systems, Strategy learning, transfer and explanation, Digital Ludeme Project, Digital archæoludology

## Abstract

This report summarises the Digital Ludeme Project, a recently launched 5-year research project being conducted at Maastricht University. This computational study of the world’s traditional strategy games seeks to improve our understanding of early games, their development, and their role in the spread of related mathematical ideas throughout recorded human history.

## Introduction

All human cultures throughout history have played games [1]. But while there exists ample archæological evidence of ancient games—typically game boards and pieces—the rules for actually playing these games are not always known, creating huge gaps in our knowledge of this important part of our cultural heritage.

The Digital Ludeme Project^1^ is a 5-year research project being conducted at Maastricht University over 2018–2023, funded by a European Research Council (ERC) Consolidator Grant. The objectives of the project are to:

1. *Model* the full range of traditional strategy games in a single, playable digital database.
2. *Reconstruct* missing knowledge about traditional strategy games with an unprecedented degree of accuracy.
3. *Map* the development of traditional strategy games and explore their role in the development of human culture and the spread of mathematical ideas.

An ultimate goal of the project is to produce a “family tree” of the world’s traditional strategy games, with which the dispersal of games and related mathematical ideas might be traced throughout recorded history. *Traditional strategy games* are those with no proprietary owner [2, p. 5] that exist in the public domain, and in which players succeed through mental rather than physical acumen. This study will cover the full range of such games throughout recorded human history, i.e. from around 3500bc, from all countries and cultures worldwide. This paper gives a brief overview of this project, which is still in its early stages, with an emphasis on relevant AI aspects.

*Research context* While there is much archæological evidence of ancient games, the rules for playing them are usually lost [3] and must be reconstructed by historians according to their knowledge of the cultures in which they were played [4, 5]. While there has been considerable historical research into games and their use as tools of cultural analysis, much is based on the interpretation of partial evidence with little mathematical analysis, and our modern understanding of ancient games is based on (unreliable) modern reconstructions.

**Fig. 1:**
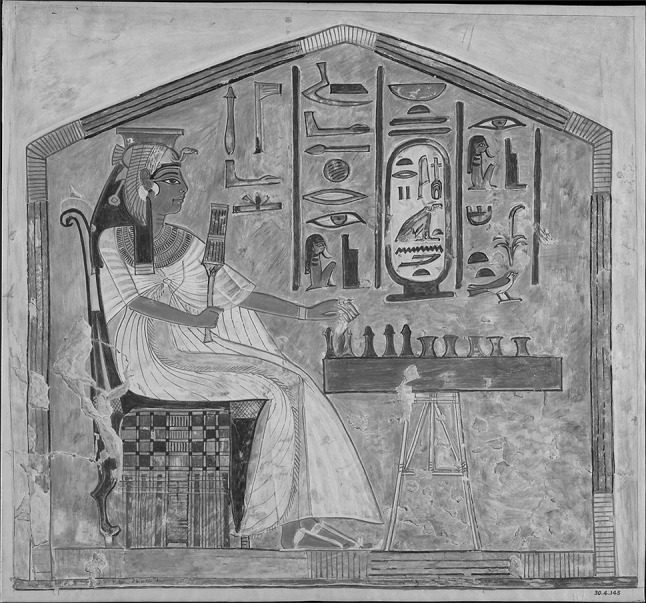
Queen Nefertari playing Senet (c.1279–1213bc)

For example, Fig. 1 shows ancient Egyptian hieroglyphic art depicting Queen Nefertari playing Senet, one of the first known board games, c.1279–1213bc.^2^ While many copies of Senet have been found dating back over 5000 years—including complete sets with board and pieces in pristine condition—no written account of how the game was actually played has ever been found. Historians have had to piece together probable reconstructions from clues found in hieroglyphic art and their knowledge of ancient Egyptian culture, and the game is played today according to a variety of contrasting rule sets.

The literature abounds with examples of plausible reconstructions that have later proven flawed due to translation errors, transcription errors, bad assumptions, crippling oversights, etc., stemming from a lack of appropriate mathematical analysis. The archæological record of ancient games has the potential to offer valuable insights into this aspect of our cultural heritage, and allow useful comparative cultural analyses, but not until the appropriate tools are developed to allow a greater degree of mathematical rigour.

## Modelling Games

The project will involve a comparison of the world’s 1000 most influential traditional strategy games throughout recorded history. This requires a model capable of describing the full range of games in a single consistent format.

*Ludemes* Games are modelled as structures of *ludemes*, i.e. game memes or conceptual units of game-related information [6]. These constitute a game’s underlying building blocks and distinguish between its *form* (rules and equipment) and *function* (behaviour that emerges through play) to provide a clear genotype/phenotype analogy. Ludemes are the high-level conceptual terms that human designers use to describe games, which make games easier to model, compare and manipulate digitally.

For example. Table 1 shows the game of Tic-Tac-Toe in ludemic form. This description is simple, clear, encapsulates key concepts and labels them with meaningful names. Breaking games down into ludemes makes them easier to model, compare and manipulate digitally, and makes it possible the model the full range of traditional games in a single playable database.

**Table 1:**
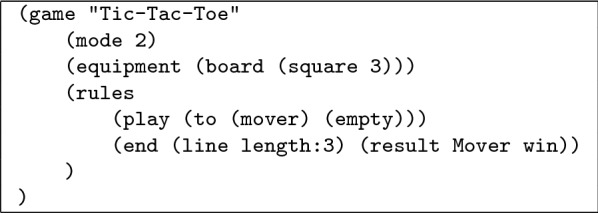
Ludemic description of Tic-Tac-Toe

### The Ludii General Game System

The ludemic model forms the basis of a new *general game system* (GGS) called Ludii that will be able to play, evaluate and optimise and sufficiently wide range of games. It builds on my earlier Ludi system [7] but offers much greater generality and extensibility due to a new *class grammar* approach [8] which compiles ludemic descriptions directly into executable code. The programming language (Java) effectively becomes the GDL, allowing the definition of almost any known ludeme for traditional games of strategy.

*MCTS move planning* AI move planning will be performed using *Monte Carlo tree search* (MCTS) [9] with playouts biased by features learnt through self-play. MCTS has become the preferred approach for general game playing over recent years, due to its ability to devise plausible actions in the absence of domain knowledge about the given task. It can prove weak for some games, but generally provides a good baseline level of AI play for most games.

The combination of deep learning with MCTS has recently had spectacular success with Go [10]. However, this level of superhuman performance is not required for this project, where a more modest level of play pitched just beyond average human level is preferable, in order to estimate the potential of games to interest human players. Superhuman AI that plays differently to humans could actually bias evaluations; instead, we want an AI that makes moves that human players would plausibly make.

**Fig. 2:**
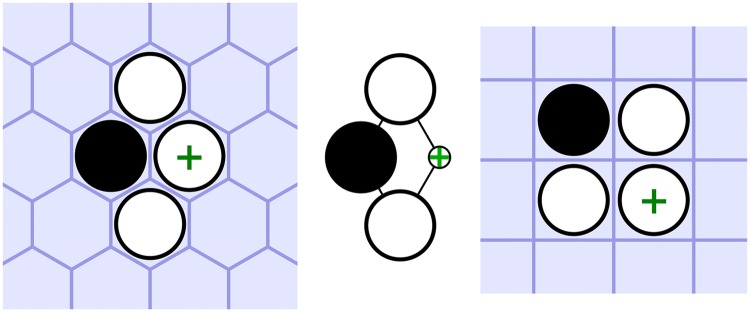
A strong pattern for hexagonal connection games transferred to a square grid

*Strategic features* MCTS playouts are biased by lightweight features representing local geometric piece patterns. These are based on the adjacency of the board underlying graph, rather than being tied to any one particular board topology, to facilitate the transfer of learnt features readily between different board types. For example, Fig. 2 shows a pattern known to benefit connection games played on the hexagonal grid [11] (left) transferred to a square grid (right). The “+” indicates a good move to make when this pattern is found.

It is hypothesised that such piece patterns encode local strategies relevant to the game being played. If true, then this provides a potential metric with which to measure the full range of games for *quality* (i.e. their potential to interest human players) for evaluating reconstructed rule sets.

Lantz et al. propose the *strategy ladder* [12] and suggest that the most interesting games are those in which players are able to immediately learn some basic strategies as they play the game, and continue to learn increasingly complex strategies the more they play it. This seems most appropriate for the games being studied in this project, i.e. traditional *strategy* games.

## Reconstruction

We aim to produce better reconstructions of rule sets for traditional strategy games that maximise both: (1) the historical *authenticity* of rule sets as cultural artefacts, and 2) their *quality* as games.

*Genetics of games* In order to map the dispersal of traditional strategy games, it is useful to cast the mechanism for their evolution into a biological genetic framework. Anthropologist Alex de Voogt has stated: *There is nothing genetic about board games. There are no genes or mental parameters that only change with a new generation of people as in linguistics or in biology* [13, p. 105]. However, the ludemic model allows us to distinguish between the *form* of a game defined by its ludemic makeup of rules and equipment (i.e. genotype) and the *function* of a game defined by the behaviour it exhibits when played (i.e. phenotype). Ludemes are the “DNA” that define each game, and the ludemic approach has already proven to be a valid and powerful model for evolving games [7].

*Computational phylogenetics* Once a genetic framework has been established, *computational phylogenetics* techniques such as those used to create phylogenetic trees mapping the dispersal of human language [14] can be applied. Such techniques allow *ancestral state reconstruction* for estimating the likelihood of given traits occurring in “ancestor” games, and the inference of possible *missing links* in the form of unknown games suggested by the phylogenetic record for which no evidence exists.

Phylogenetic techniques have previously been applied to subsets of Mancala games [15] and Chess-like games [16]. However, phylogenetic analyses of such cultural domains tend to confuse the genotype and phenotype of artefacts, yielding classifications of questionable value based on superficial traits rather than meaningful underlying structures [17]. List et al. provide guidelines for correctly casting cultural domains in a biological framework [18].

*Game distance* Games do not contain the traces of genetic heritage that biological organisms do; rule sets are typically optimised and superfluous rules stripped out, making their heritage hard to trace. In lieu of a metric for genetic distance, the *ludemic distance* between games will be used, given by the *weighted edit distance* (WED) between ludemic descriptions, i.e. the number of removals, insertions and edits required to convert one into the other, weighted according to the relative importance of each attribute. This is similar in principle to the *Hamming distance* used to quantify the similarity between DNA sequences in bioinformatics [19]. Care must be taken to detect and handle *homologies* [20] that occur when different ludeme structures produce the same behaviour in play.

**Fig. 3:**
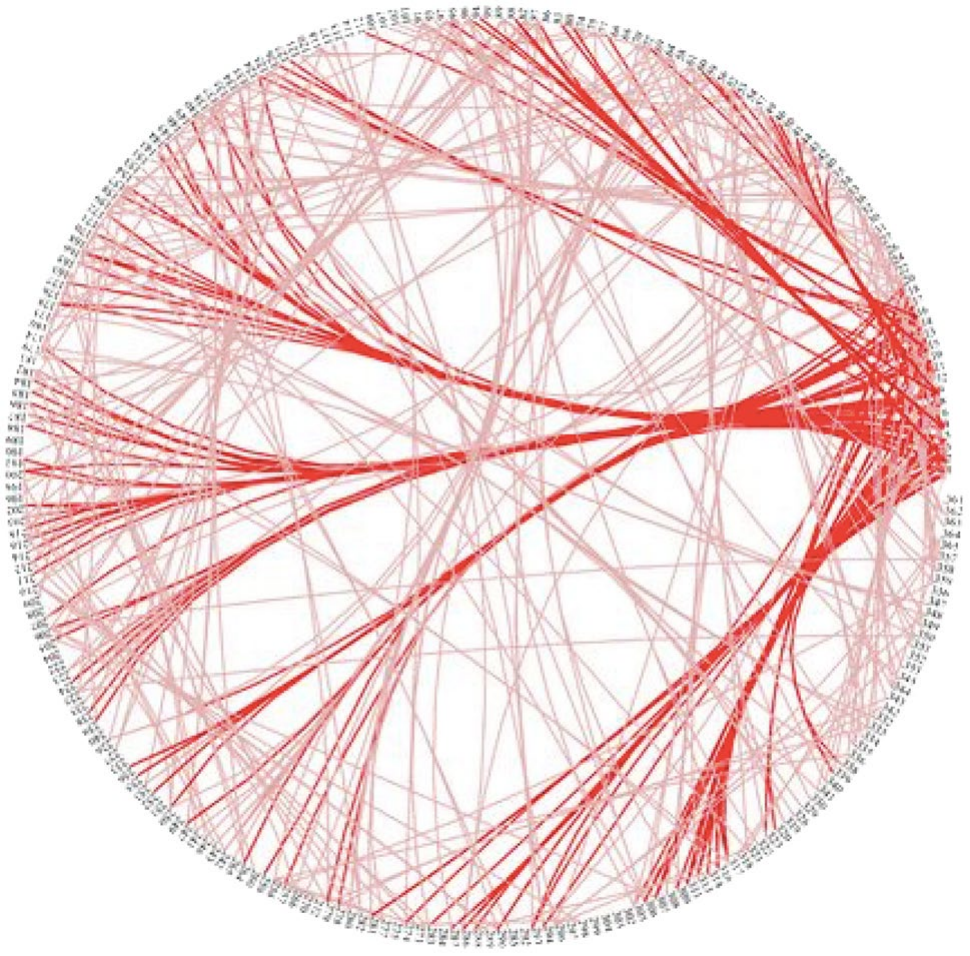
Horizontal influence map showing connections between programming languages [23]

*Horizontal influence maps* Morrison points out that phylogenetic *networks* may be more suitable than *trees* for modelling the evolution of cultural artefacts [21]. This seems especially relevant for games, which are more likely to have evolved through distributed *polygenesis* from multiple sources than *monogenesis* from a single common ancestor [22], and in which rules can pass from one to another through *ethnogenesis* (i.e. horizontal gene transfer) rather than classic inheritance. The prevalence of ethnogenesis in the spread of games could warrant the use of *horizontal influence maps* (HIM) [23] rather than standard phylogenetic approaches based on vertical gene transfer. For example, Fig. 3 shows HIM analysis of connections between programming languages revealing a new perspective on their historical development (each labelled node on the perimeter of the map denotes a programming language).

## Mapping

Ludeme classes will be tagged with keywords indicating the underlying mathematical concepts that they embody, and game descriptions in the game database will be tagged with details regarding when and where they were played (among other historical/cultural details). Each game will therefore have a *mathematical profile* based upon its component ludemes and a *historical profile*. The game database will be data-mined for common ludemeplexes that represent important game mechanisms. The associated metadata will be cross-referenced to create *knowledge graphs* that give probabilistic models [24] of the relationships between their geographical, historical and mathematical dimensions.

The cultural location of games will be achieved using a geo-location service such as GeaCron.^3^ GeaCron maintains a database of geo-political world maps for every year from 3000bc to the present day, which can be queried to specify which empire, nation, civilisation or culture correlates with any given geographical location in recorded history. GeaCron also provides details of known trade routes, expeditions, and other key historical events. This provides a mechanism for correlating the spread of games, ludemes and associated mathematical ideas with the spread of human civilisation.

## Digital Archæoludology

This project is pioneering a new field of study called *digital archæoludology* (DAL) which involves the analysis and reconstruction of traditional games from incomplete descriptions using modern computational techniques [25]. The aim is to provide tools and methods to help game historians and researchers better understand traditional games and their development.

Traditional game studies have tended to focus on the authenticity of reconstructions (as cultural artefacts) rather than their actual quality as games. DAL seeks to redress this imbalance by searching for plausible reconstructions that maximise both quality and historical authenticity, hopefully leading to better reconstructions and a better understanding of ancient and early games.

## Conclusion

The Digital Ludeme Project is still in its early stages. Work is currently focussed on developing the Ludii game system, then will move on to populating the full game database and performing the phylogenetic and cultural mapping tasks over the next few years. Its aim is to use modern computational techniques to provide tools and techniques for helping to fill the gaps in our knowledge of traditional strategy games and their development.

## Footnotes

1 http://www.ludeme.eu.

2 New York Metropolitan Museum: https://www.metmuseum.org/art/collection/search/548355 (CCO 1.0).

3 http://geacron.com.
